# Debye formulas for a relaxing system with memory

**DOI:** 10.1038/s41598-018-21028-2

**Published:** 2018-02-19

**Authors:** Ivo Klik, James McHugh, Roy W. Chantrell, Ching-Ray Chang

**Affiliations:** 1 Department of Physics, National Taiwan University, Taipei, Taiwan; 2 Department of Physics, The University of York, York, YO10 5DD UK

## Abstract

Rate (master) equations are ubiquitous in statistical physics, yet, to the best of our knowledge, a rate equation with memory has previously never been considered. We write down an integro-differential rate equation for the evolution of a thermally relaxing system with memory. For concreteness we adopt as a model a single-domain magnetic particle driven by a small ac field and derive the modified Debye formulas. For any memory time Θ the in-phase component of the resultant ac susceptibility is positive at small probing frequencies *ω*, but becomes negative at large *ω*. The system thus exhibits frequency induced diamagnetism. For comparison we also consider particle pairs with dipolar coupling. The memory effect is found to be enhanced by ferromagnetic coupling and suppressed by antiferromagnetic coupling. Numerical calculations support the prediction of a negative susceptibility which arises from a phase shift induced by the memory effect. It is proposed that the onset of frequency induced diamagnetism represents a viable experimental signature of correlated noise.

## Introduction

The Debye formulas determine the initial ac response of a two level, equilibrium system to an applied ac perturbation. We address here the dynamic magnetic susceptibility discussed by *e.g*. Bermúdez *et al*^1^. or Klik *et al*.^2^. Motivating the research is the underlying assumption of uncorrelated (white) noise adopted in all models to date and the need to investigate the implications of colored noise arising from the temporal correlation of heat bath properties. This is an important generalization of the theoretical approach and is especially timely in relation to the current advances in ultrafast laser processes involving femtosecond laser processes. Following the pioneering work of Beaurepaire *et al*.^3^ demonstrating ultrafast demagnetization of Ni, more recent work has shown extremely complex and subtle behavior during laser driven processes including thermally driven magnetization switching^4,5^ in which the magnetization is reversed by the action of a thermal pulse in the absence of an applied field. Ultrafast experiments rely on the rapid thermally driven relaxation of the spin system, modelled in refs^4,5^. using a white noise thermostat, however it has been shown^6^ that correlated noise can significantly slow down the relaxation processes. Ultrafast laser processes are extremely complex, for this reason we consider here the study of the effects of correlated noise on the well-understood Debye formulas. This provides an important generalization of the Debye formulas and also provides an experimentally realizable means of determine the correlation times.

For simplicity we adopt the well known uniaxial single-domain particle with magnetic field applied parallel to its easy axis. The corresponding free energy^7^ is

1 $$E/KV=1-2hx-{x}^{2}$$

with *K* the anisotropy constant, *V* the particle volume and the reduced field *h* = *H*/*H*_*K*_ where *H* = *H*(*t*) is the time dependent applied magnetic field and *H*_*K*_ = 2*K*/*M*_*s*_ is the anisotropy field. Further, *M*_*s*_ is the saturation magnetization of the particle and *x* = cos*ϑ*, where *ϑ* is the angle spanned by the magnetization vector and the applied field.

At $|h| < 1$ the system has two local minima located at *x* = ±1 and thermally activated transitions between these two minima take place with rates ${\kappa }_{12}={\kappa }_{1\to 2}$ and ${\kappa }_{21}={\kappa }_{2\to 1}$. Neglecting all intrawell processes^8–11^, the thermally activated dynamics of the system are then given by the simple master (rate) Eq.^7,12^ for the occupation probabilities *n*_1_ and *n*_2_ = 1 − *n*_1_,

2 $${\dot{n}}_{1}=-{\kappa }_{12}{n}_{1}+{\kappa }_{21}{n}_{2}=-({\kappa }_{12}+{\kappa }_{21}){n}_{1}+{\kappa }_{21},$$

${\dot{n}}_{1}=d{n}_{1}/dt$ and we made use of the probability normalization. For the uniaxial particle (1) there is ${\kappa }_{12}={f}_{0}\exp [-q{\mathrm{(1}+h)}^{2}],$ and ${\kappa }_{21}={f}_{0}\exp [-q{\mathrm{(1}-h)}^{2}]$ where *f*_0_ is the prefactor, usually taken to be a constant^13,14^ and $q=KV/{k}_{B}T$ where *k*_*B*_ is the Boltzmann constant and *T* the ambient temperature. Let now

3 $$h(t)={h}_{0}\,\cos \,\omega t$$

where 0 < *h*_0_ ≪ 1 is a small field amplitude and *ω* is the field frequency. Solving Eq. (2) in the first order of *h*_0_ we obtain for the reduced magnetization *m* = *M*/*M*_*s*_ the expression

4 $$m={n}_{1}-{n}_{2}={h}_{0}({\chi }_{1}\,\cos \,\omega t+{\chi }_{2}\,\sin \,\omega t)+O({h}_{0}^{2}),$$

with the Debye susceptibilities

5 $${\chi }_{1}(\omega )=8q{\tilde{n}}_{\uparrow \uparrow }\frac{{{\rm{\Gamma }}}^{2}}{{\omega }^{2}+{{\rm{\Gamma }}}^{2}},$$

6 $${\chi }_{2}(\omega )=8q{\tilde{n}}_{\uparrow \uparrow }\frac{\omega {\rm{\Gamma }}}{{\omega }^{2}+{{\rm{\Gamma }}}^{2}},$$

Γ = 2*f*_0_*e*^−*q*^. In these expressions we introduced the quantity ${\tilde{n}}_{\uparrow \uparrow }=\mathrm{1/4}$ whose significance shall become apparent later, when we consider the response of interacting particle pairs. It should be noted that we use only *O*(*h*_0_) terms of the relaxation rates whose precise form is thus irrelevant. For this reason Eqs (5) and (6) hold also for other physical quantities, such as electric susceptibility^1^. At this point is is worth evaluating the generality of the master equation expressions for the susceptibility. To do this we have evaluated the temperature dependence of the susceptibility in Supplementary Information S1. It is shown that Eqs (5) and (6) predict results in good agreement with experiment, with correct limiting behaviour as $q\to \infty$ and $q\to 0$. In Supplementary Information S1 we also show that the numerical approach, to be described shortly, give behaviour in good qualitative agreement.

If the system has memory, then in place of Eq. (2) we write the integro-differential equation

7 $${\dot{n}}_{1}=-{\int }_{0}^{t}K(t-\tau )[{\kappa }_{12}(\tau )+{\kappa }_{21}(\tau )]{n}_{1}(\tau )d\tau +{\int }_{0}^{t}K(t-\tau ){\kappa }_{21}(\tau )d\tau $$

with the memory kernel *K*(*t*), which we take to have the exponential form^15,16^

8 $$K(t)=\frac{{e}^{-|t|/{\rm{\Theta }}}}{2{\rm{\Theta }}}\to \delta (t)\,\,as\,{\rm{\Theta }}\to \mathrm{0,}$$

Θ is the memory time. This topic has been discussed in a different context by Sun *et al*.^17,18^.

To first order in *h*_0_ Eq. (7) is composed of two convolution integrals and is readily solved. The long time, steady state solution then has the form (4), with

9 $${\chi }_{1}(\omega )=8q{\tilde{n}}_{\uparrow \uparrow }\frac{{\rm{\Gamma }}({\rm{\Gamma }}-{\omega }^{2}{\rm{\Theta }})}{{\omega }^{2}+{({\rm{\Gamma }}-{\omega }^{2}{\rm{\Theta }})}^{2}},$$

10 $${\chi }_{2}(\omega )=8q{\tilde{n}}_{\uparrow \uparrow }\frac{\omega {\rm{\Gamma }}}{{\omega }^{2}+{({\rm{\Gamma }}-{\omega }^{2}{\rm{\Theta }})}^{2}}\mathrm{.}$$

**Figure 1:**
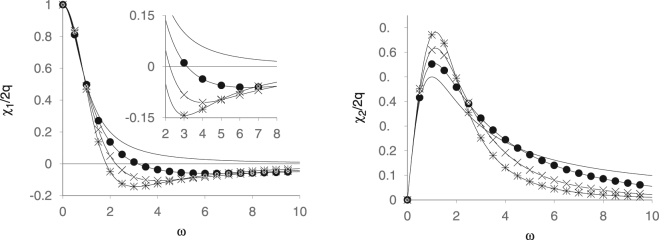
The in-phase (left) and out-of-phase (right) components of the ac susceptibility versus the frequency *ω* of the probing field. The rate Γ = 1 Hz and the memory time Θ = 0 s (unmarked curves), 0.1 s (•), 0.2 (s) (×) and 0.3 s (*). The interaction strength *ρ* = 0. The inset depicts a close-up of the in-phase susceptibility.

The two functions *χ*_1_(*ω*) and *χ*_2_(*ω*) are plotted in Fig. 1, where we note we have used a large value of the escape rate, Γ = 1 *Hz*, for visual clarity. The salient feature of the in-phase component *χ*_1_ is the fact that it becomes negative at sufficiently large frequencies for any Θ > 0, passes through a local minimum and only then rapidly approaches its asymptotic zero value from below. This means that the particle is paramagnetic at low frequencies, but diamagnetic at high frequencies. In the diamagnetic phase of the response the particle is apparently slowed down by the medium in which it finds itself and cannot follow the rapidly oscillating field. One can therefore expect to find the frequency induced diamagnetism in particles suspended in viscous fluids. However, the effect has previously been observed experimentally in single crystals by Rhyee *et al*.^19^. Theoretically the effect can be so strong that it would render the magnetic permeability negative^20^. The out-of-phase component is interesting in that its peak grows significantly higher and narrower with increasing memory time, so that we conclude that memory enhances energy losses and heating of the sample in a narrow frequency interval. This behavior is required from particles used to treat cancer by magnetic particle hyperthermia^21^. We note that, in the fully hysteretic regime with a conventional (white noise) thermostat, El Mrbati *et al*.^22^ have shown diamagnetic behaviour, here we demonstrate a very general effect arising from the nature of the noise itself.

So far we have considered only noninteracting media. Now we shall consider an exactly solvable model system of two identical superparamagnetic particles with parallel easy axes^7,23^. The particles interact with each other via dipolar coupling and have three metastable states: $\uparrow \uparrow$, $\uparrow \downarrow +\downarrow \uparrow$ and $\downarrow \downarrow$. The mutual coupling is ferromagnetic if the bond angle *β* between the easy axes is zero, *β* = 0 and antiferromagetic if *β* = *π*/2. The thermally driven dynamics of the particle pair, at the *small* fields of interest here, are described by the three level master equation^7^

11 $${\dot{n}}_{1}=-\mathrm{(2}{\kappa }_{12}+{\kappa }_{21}){n}_{1}-{\kappa }_{21}{n}_{3}+{\kappa }_{21},$$

12 $${\dot{n}}_{3}=-{\kappa }_{23}{n}_{1}-\mathrm{(2}{\kappa }_{32}+{\kappa }_{23}){n}_{3}+{\kappa }_{23},$$

where again we made use of the probability conservation *n*_1_ + *n*_2_ + *n*_3_ = 1. The two particle occupation probabilities are ${n}_{1}={n}_{\uparrow \uparrow }$, ${n}_{2}={n}_{\uparrow \downarrow +\downarrow \uparrow }$ and ${n}_{3}={n}_{\downarrow \downarrow }$. Obviously, *n*_2_ corresponds to a demagnetized state and the reduced magnetization *m* = 2(*n*_1_ − *n*_3_). The rates of the thermally activated transitions between the individual minima, ${\kappa }_{ij}={\kappa }_{i\to j}$, are given by the formula

13 $${\kappa }_{ij}={f}_{0}{e}^{-q{Q}_{ij}},$$

with

14 $${Q}_{12}={h}_{c}^{2}+\varepsilon \rho +2h\mathrm{(2}-{h}_{c}),$$

15 $${Q}_{21}={h}_{c}^{2}-2h{h}_{c},$$

16 $${Q}_{23}={h}_{c}^{2}+2h{h}_{c},$$

17 $${Q}_{32}={h}_{c}^{2}+\varepsilon \rho -2h\mathrm{(2}-{h}_{c}\mathrm{).}$$

The dipole strength $\rho ={M}_{s}^{2}V\mathrm{/2}K{R}^{3}$ where *R* is the distance between the particles. If the bond angle *β* = 0, then the critical field ${h}_{c}^{2}\mathrm{=(1}-\rho \mathrm{)(1}-3\rho )$ and *ε* = 8. At *β* = π/2 there is ${h}_{c}^{2}\mathrm{=(1}-\rho \mathrm{)(1}+3\rho )$ and *ε* = −4. Introducing now memory into Eqs (11) and (12) in the same manner as was done in Eq. (7) and linearizing with respect to *h*_0_, we obtain again the formulas (9) and (10), but with ${\rm{\Gamma }}=2{f}_{0}{e}^{-q({h}_{c}^{2}+\varepsilon \rho )}$, and

18 $${\tilde{n}}_{\uparrow \uparrow }=\mathop{\mathrm{lim}}\limits_{t\to \infty }{n}_{1}(t)=\frac{1}{\mathrm{2(1}+{e}^{-\varepsilon \rho })}$$

in zero field. The formal equivalence of the two results, for interacting and noninteracting particles, follows from the fact that in the first order of *h*_0_ Eqs (11) and (12) yield a single rate equation for the reduced magnetization *m*. This property follows from the symmetry of the interacting system and is preserved also if a mean field theory is employed. However, it could not hold for more complex, strongly coupled particle assemblies whose response is thus not of the Debye type^24^. It should be noted that ferromagnetic coupling with *ε* > 0 reduces the rate Γ and facilitates the onset of the diamagnetic state, while antiferromagnetic coupling, which favors the creation of magnetically neutral demagnetized states, has the opposite effect.

The dynamics of superparamgnets are also amenable to investigation via direct numerical simulation of the corresponding Langevin equation, the Landau-Lifshitz-Gilbert (LLG) equation. The Markovian rate equations of the form Eq. 2 correspond to the high barrier limit of the LLG^14,25^, in what is termed the discrete orientation approximation. The preceding analysis of the non-Markovian rate equations may then be understood as the direct modification of said high barrier rate equations to include explicit memory effects. In what follows we will compare this approach to that of an explicitly non-Markovian Langevin equation for the magnetic system.

The LLG equation for the time-evolution of a single spin is

19 $$\frac{d{\bf{S}}}{dt}=-\frac{\gamma }{1+{\alpha }^{2}}({\bf{S}}\times {\bf{H}}+\alpha {\bf{S}}\times ({\bf{S}}\times {\bf{H}})),$$

where *α* is the phenomenological damping constant, $\gamma =1.76\times {10}^{11}\,{T}^{-1}\,{s}^{-1}$ is the gyromagnetic ratio and S is a unit vector representing the direction of the magnetic moment. The local magnetic field, **H**, is obtained from the spin Hamiltonian, ${\mathcal H}$, as ${\bf{H}}=-\frac{1}{{\mu }_{s}}\frac{\partial  {\mathcal H} }{\partial {\bf{S}}}$, where *μ*_*s*_ is the spin moment. This may include anisotropy, exchange and other fields as appropriate. At non-zero temperature the local field is augmented by a thermal field, **H**_*th*_, whose components are assumed to be spatially and temporally uncorrelated, thus

20 $$\langle {H}_{th,i}(t)\rangle =0$$

21 $$\langle {H}_{th,i}(t){H}_{th,j}(t^{\prime} )\rangle =\frac{2\alpha {k}_{B}T}{\gamma {\mu }_{s}}\delta (t-t^{\prime} ){\delta }_{ij},$$

where (*i, j*) refer to Cartesian coordinates.

The Landau-Lifshitz-Miyazaki-Seki (LLMS) Eq.^6^, is the natural non-Markovian extension of the LLG, via the introduction of time-correlated, coloured noise, Under this approach, the spin no longer includes an explicit damping term; rather we consider a spin undergoing only precessional motion. Damping and correlations are included via a bath magnetic field with a characteristic correlation time and which dynamically evolves to align with the spin while also being driven by a white noise. The time evolution of the spin and the bath field are of the form^6^

22 $$\frac{d{\bf{S}}}{dt}=\gamma ({\bf{S}}\times ({\bf{H}}+{\eta }))$$

23 $$\frac{d{\eta }}{dt}=-\frac{1}{{\rm{\Theta }}}({\eta }-\lambda {\bf{S}})+{\bf{R}},$$

where Θ is the noise correlation time, *λ* is the coupling of the bath to the spins and *η* is the correlated thermal magnetic field. The fluctuation-dissipation theorem for the white noise **R** is

24 $$\langle {R}_{i}(t){R}_{j}(t^{\prime} )\rangle =\frac{2\lambda {k}_{B}T}{{\mu }_{s}{\rm{\Theta }}}\delta (t-t^{\prime} ){\delta }_{ij}\mathrm{.}$$

The Langevin equation for the spin-only is

25 $$\frac{d{\bf{S}}(t)}{dt}=\gamma {\bf{S}}(t)\times [{\bf{H}}+\bar{{\bf{R}}}(t)-\lambda {\int }_{-\infty }^{t}dt^{\prime} \psi (t-t^{\prime} )\frac{d{\bf{S}}(t^{\prime} )}{dt^{\prime} }],$$

where $\psi (t)={e}^{-t/{\rm{\Theta }}}$, $\bar{{\bf{R}}}(t)$ is exponentially-correlated thermal noise $\langle {\bar{R}}_{i}(t){\bar{R}}_{j}(t^{\prime} )\rangle =\frac{\lambda {k}_{B}T}{{\mu }_{s}}\psi (t-t^{\prime} ){\delta }_{ij}$ and the last term represents the damping which contains an effect of retardation due to the finite relaxation time of the fluctuating field. The spin-only LLMS is very similar to the LLG equation in the form $\frac{d{\bf{S}}}{dt}=-\gamma ({\bf{S}}\times {\bf{H}}-\alpha {\bf{S}}\times \frac{d{\bf{S}}}{dt})$ and by taking the ${\rm{\Theta }}\to 0$ limit, we may recover the LLG equation and obtain the relation *α* = *γλ*Θ. Both sets of equations are integrated using the Heun method which is consistent with the Stratonovich calculus.

To calculate the susceptibility, we simulate the time evolution of an ensemble of non-interacting spins using either LLG or LLMS as appropriate. The spin is first evolved in time until the system has thermalised. Following this an oscillating magnetic field *h*_*z*_ = *h*_0_cos(*ωt*) was applied to the system along the anisotropy direction, where *h*_0_ is the field magnitude and *ω* the frequency. The real and imaginary susceptibility, respectively *χ*_1_ and *χ*_2_ are calculated as the cosine and sine Fourier transform of 〈*S*_*z*_〉, the ensemble average of the *z*-component of the spin. Calculations as a function of ac field magnitude were carried out to ensure the model was applied in the linear region of the system response.

For the following simulations we consider an ensemble of non-interacting Co nanoparticles in the absence of external fields. The particles are assumed to have a volume of *V* = 8 × 10^−27^ *m*^3^, anisotropy energy *KV* = 3.36 × 10^−21^ *J* and a magnetic moment of *μ*_*s*_ = 1.12 × 10^−20^ *J/T*. The temperature and damping parameters are taken such that *q* = 2 and *α* = 0.5. An ensemble of 10,000 spins were used for the simulations. We are especially interested in the behaviour of the real part of the dynamic susceptibility, *χ*_1_ in the vicinity of the analytically-predicted diamagnetic phase transition.

**Figure 2:**
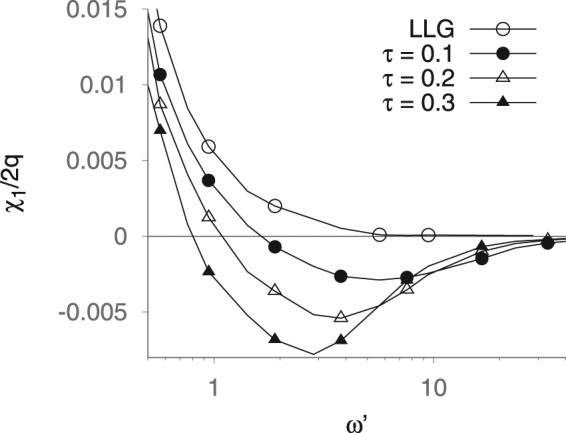
The variation of the real part of the susceptibility with the frequency, normalised by the Larmor frequency, $\omega ^{\prime} =\omega /\gamma {H}_{k}$) for various values of $\tau =(\gamma {H}_{k}{)}^{-1}{\rm{\Theta }}$, in the vicinity of the diamagnetic phase transition.

The variation of the real part of the susceptibility with frequency (normalised by the Larmor frequency, $\omega ^{\prime} =\omega {(\gamma {H}_{k})}^{-1}$) for various values of *τ* = Θ*γH*_*k*_ for high frequencies, on the order of the expected phase transition, is shown in Fig. 2. This shows that the susceptibility with correlations becomes negative in this region, supporting the analytical predictions of the master equation approach and confirming that the negative susceptibility arises from a phase change induced by the slowing down of the response of the particle by the medium. We also note a cross-over in the variation of *χ*_1_ with frequency, in agreement with the analytical predictions. Overall, the numerical calculations represent an important counterpart to the analytical model. The presence of a diamagnetic phase transition under both approaches leads us to anticipate that such a transition may be the generic result of memory in magnetic systems, regardless of the precise form of the memory kernel.

**Figure 3:**
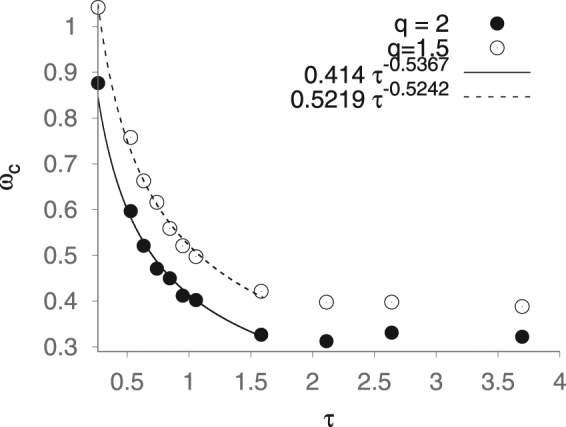
Estimated critical frequency, *ω*_*c*_ of diamagnetic phase transition vs correlation time and numerical fit for low *τ*. The *τ*-dependence compares favourably to the prediction of the master equation, ${\omega }_{c}\propto {\tau }^{-1}$ in this regime. The prefactor is similarly comparable to $\sqrt{2{e}^{-q}}$ at low *τ*.

Finally, We consider the critical frequency for the transition to diamagnetic behaviour. From Eq. 9 the transition to diamagnetic behavior occurs at the frequency ${\omega }_{c}^{2}={\rm{\Gamma }}/{\rm{\Theta }}\propto {\tau }^{-1}$. Thus we estimate that

26 $${\rm{\Theta }}=2{f}_{0}\exp -(KV/kT)/{\omega }_{c}^{2},$$

relating the measurable critical frequency to the correlation time Θ. In Fig. 3 we show the correlation time dependence as exhibited by the LLMS and see that the low-*τ* behaviour coincides with the master equation, varying as 1/√τ. The prefactor likewise compares well to $\sqrt{2{f}_{0}{e}^{-q}}$, as the Larmor frequency is a good first approximation to the attempt frequency, so we may take *f*_0_ = 1 in the reduced units and the anticipated prefactor is then simply $\sqrt{2{e}^{-q}}=\mathrm{0.668,}\,0.52$ for *q* = 1.5, 2 respectively.

Overall, the behavior predicted by the LLMS approach shows non-monotonic behavior with an increase for large Θ. We anticipate that this departure is due to the assumption of a fixed transition rate, Γ, between the wells in the master equation expression, an assumption which is not generally valid for the LLMS model, where the transition rate will be some, potentially complicated, function of Θ and *λ*. Given that we have maintained a fixed phenomenological damping, *α* = *γλ*Θ, an increase in the correlation time will cause a corresponding decrease in the coupling of the bath and the spin. The effect of the low-*λ* limit on the escape dynamics is much the same as the low-*α* behaviour of the LLG and will generally lead to a decrease in the escape rate in the limit of vanishing *λ*. Similarly, an increase in the correlation time of the thermal field will tend to decrease the transition rate between wells^26^. This effect is similar to the increase in the longitudinal and transverse relaxation times for exchange-coupled spins undergoing LLMS evolution^6^.

In summary, we have presented analytical and numerical studies of the frequency dependent susceptibility of magnetic nanoparticles. Remarkably, the calculations, consistently between the analytical and numerical models, show a frequency-induced diamagnetic susceptibility above a critical frequency dependent on the correlation time. The effect is shown to be enhanced by the presence of interparticle interactions, leading to the intriguing suggestion that interaction effects themselves may be considered as a memory effect. It should be noted that further work is needed to link the LLMS approach to the exponentially correlated noise introduced into the master equation formalism. However, this is a non-trivial problem which can only be achieved via the Fokker-Planck equation and is beyond the scope of the current work. However, we stress that the negative susceptibility arises from a phase shift induced by the low frequency filtering property of the non-Markovian system for both distinct models. Thus it is suggested that the negative susceptibility will be a common feature of systems with correlated noise. The effects of heat-bath correlations are not easy to investigate experimentally. Our prediction of frequency-induced diamagnetic behavior represents an important prediction of a clear experimentally accessible signature of heat-bath correlations, with Eq. 26 giving a direct relationship between the correlation time and the critical frequency for diamagnetic behaviour. Measurements of the correlation time would generate new understanding of the properties of thermal baths and their interaction with spin systems and would be especially important in the understanding of ultrafast magnetization processes as pointed out in ref.^6^.

## Electronic supplementary material

Supplementary Information
