# Supplementary material for: Debye formulas for a relaxing system with memory
Source: Sci Rep. 2018 Feb 19;8:3271. doi: 10.1038/s41598-018-21028-2 (PMC5818502; doi:10.1038/s41598-018-21028-2)
Supplement: Supplementary file 1 — Supplementary Information [file 41598_2018_21028_MOESM1_ESM.pdf]

Debye formulas for a relaxing system with memory - Supplementary Information

Ivo Klik[1], James McHugh[2,*], Roy W. Chantrell[2], and Ching Ray Chang[1]

[1]Department of Physics, National Taiwan University, Taipei, Taiwan
[2]Department of Physics, The University of York, York, YO10 5DD, UK
[*]james.mchugh@york.ac.uk

S1: Temperature dependence of susceptibility and validity regime of the master equation approach

The paper predicts theoretically an effect not yet investigated experimentally. In order to validate both master equation and LLMS approaches we now calculate the temperature dependence of the magnetic susceptibility, which has experimentally well established features, particularly a peak in the in-phase susceptibility, and limiting behaviour such that $\chi_1 \to 0$ as $q \to 0$ and $q \to \infty$. This behaviour is a well known characteristic property of nanoparticle magnetic systems having analogous behaviour in spin glasses as first noted by Wohlfarth et.al[1]. We require that the master equation and LLMS approach reproduce this characteristic behaviour. To further emphasise the qualitative similarities between the master equation approach and the numerical LLG simulations we present a comparison of the temperature-dependence of the in-phase and out-of-phase susceptibilities from the LLG for the Co nanoparticle and

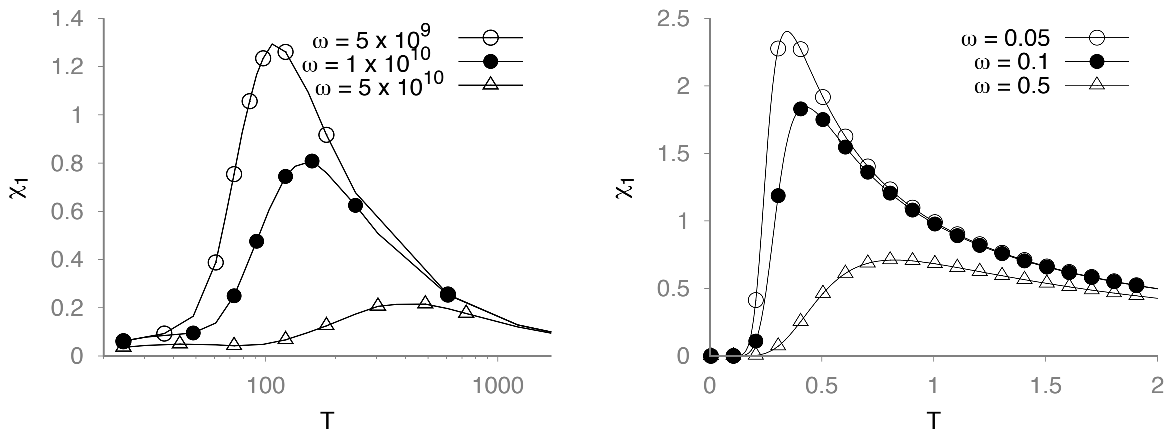

Figure S1. χ_1 vs T from (L) LLG simulations and (R) the analytical master equation.

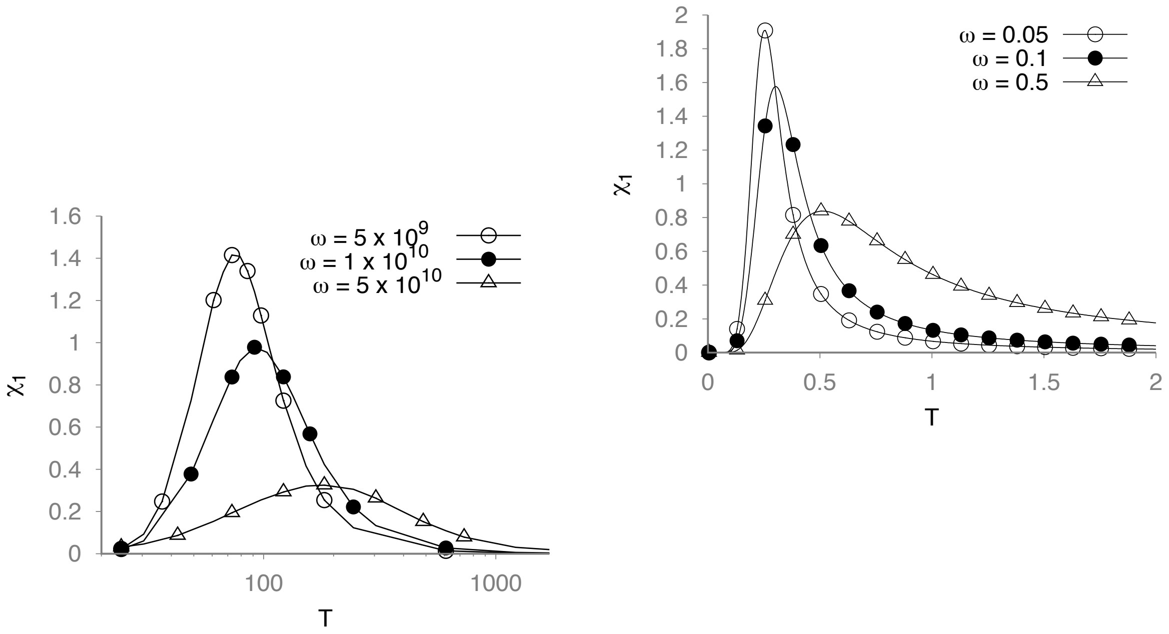

Figure S2. χ_2 vs T from (L) LLG simulations and (R) the analytical master equation.

the master equation in Supplementary Figures S1 and S2 respectively. Note the peak in the susceptibility at a frequency dependent temperature in agreement with experiment. The peak arises from a transition to thermally stable magnetic behaviour at low temperatures, where transitions over the energy barrier are of low probability, to a state where the magnetic properties are governed by thermally activated transitions leading to thermal equilibrium (superparamagnetic) states, where χ_1 decreases with increasing temperature. As shown in Supplementary Fig. S1, the peak temperature is frequency dependent, consistent with experiment[2]. Specifically, the peak arises from the transition from ferromagnetically stable behaviour with low susceptibility to the superparamagnetic regime with susceptibility $\chi_1 = \mu/3kT$. The criterion for this transition is $KV/kT = ln(tf_0)$ where t is the characteristic timescale of the measurement Limiting cases can be considered using equation (5) of the main text. In the limit $q \to 0$ we have that $\chi_1 \propto q$ and the susceptibility tends to zero as demonstrated numerically in Supplementary Fig. S1. In the low temperature limit, i.e. $T \to 0$, $\chi_1 \propto qe^{-2q}/\omega^2$, which tends to zero as $q \to \infty$, again in accordance with the calculations given in Supplementary Fig. S1. We recall from the main text that χ_1 is a reduced susceptibility, i.e. $\chi_1 = dm/dh$ with $h = H/H_K$. Thus the measured susceptibility is $\propto \mu/kT$, which is correct for our aligned case, for which the magnetisation $m = \tanh(\mu H/kT)$. Clearly χ_1 apparently diverges for the 'dc case' $\omega = 0$. Essentially this arises because at low frequency (corresponding to long measurement times) the critical temperature for the transition to superparamagnetic behaviour corresponds to $T = 0$K ($q = \infty$) at which temperature the superparamagnetic susceptibility diverges. In this case, although the master equation approach is in principle correct, the susceptibility cannot be calculated in the linear response regime which is implicit in our derivation. In practice of course this limit cannot be

accessed experimentally: even the quasi-static case of vibrating sample magnetometer measurements have a characteristic timescale $t \sim 100s$ corresponding to a measurement frequency of 0.01Hz, so our expression for non-zero frequency is generally applicable.

References

1. EP Wohlfarth, Physica **86-88B**, 852 (1977)
2. D. Fiorani. J. L. Tholence, and J . L. Dormann, Physica, **107B**, 643 (1981)
